# P-453. Cardiovascular Risk Scores and Insulin Resistance are Higher with Excess Visceral Abdominal Fat in People with HIV in the Modern Antiretroviral (ART) Era

**DOI:** 10.1093/ofid/ofae631.653

**Published:** 2025-01-29

**Authors:** Karam Mounzer, John R Koethe, Jordan Lake, Mohammed Zogheib, Colleen McGary, R Brandon Cash

**Affiliations:** Philadelphia Fight Community Health Centers, Philadelphia, Pennsylvania; Vanderbilt University Medical Center, Nashville, TN; University of Texas Health Science Center at Houston, Houston, TX; Theratechnologies, Orlando, Florida; Theratechnologies, Orlando, Florida; Theratechnologies, Inc, Montreal, Quebec, Canada

## Abstract

**Background:**

People with HIV (PWH) are at an increased risk of cardiovascular disease (CVD), even after accounting for traditional risk factors like diabetes and dyslipidemia. Metabolic syndrome, a component of which is central adiposity, is also increasingly prevalent in PWH. Yet, there are few data on the contribution of excess visceral abdominal fat (EVAF) to increased CVD risk in PWH in the modern ART era.

**Methods:**

We used the Visceral Adiposity Measurement and Observation Study (VAMOS) to investigate how EVAF impacts traditional CVD risk factors and overall CV risk in PWH. VAMOS is a cross-sectional, multi-center observational study in PWH, virologically suppressed for ≥1 year, with BMI ranging from 20 to 40 kg/m^2^. Participants completed study visits, labs, and an abdominal computed tomography scan in the overnight fasting state. Visceral fat was quantified by a single slice at L4-L5. Growth hormone (GH) levels, HOMA-IR and triglyceride:high-density lipoprotein (TG:HDL) ratios (an alternative measure of insulin resistance, IR) were also analyzed. Relationships with EVAF were assessed by Spearman correlation coefficients and Wilcoxon Two-Sample Tests (EVAF defined as ≥130 cm^2^).

**Results:**

Of the 170 participants studied, the majority were white and male with an average age of 54 years. Median visceral fat area was 148 cm^2^ (94, 218) with an EVAF prevalence of 58%. Participants with EVAF had higher HOMA-IR values (p≤.0001) and higher TG:HDL ratios (p=0.0013) than those without EVAF. There was also a positive correlation between EVAF and HOMA-IR (ρ=0.43, p≤0.0001) and TG:HDL ratio (ρ=0.33, p≤0.0001). Importantly, greater visceral fat area was associated with higher 10-year ASCVD risk (p< 0.0001). To understand a potential driver behind this relationship between EVAF and CV risk, GH levels were explored. Increasing visceral fat surface area was inversely correlated with GH levels (ρ=-0.17, p=0.03); moreover, there were lower GH levels in subjects with EVAF (p=0.05).

**Conclusion:**

Increased levels of visceral fat relate to increased CV risk scores, as well as the traditional risk factors of IR and lipid levels, which contributes to the heightened risk of CVD in PWH on modern ART regimens. The dynamics between decreased GH levels and EVAF found here support targeting EVAF reduction via the GH axis.

**Disclosures:**

**Karam Mounzer, MD**, Epividian: Board Member|Epividian: Honoraria|GS: Advisor/Consultant|GS: Grant/Research Support|GS: Honoraria|Merck: Advisor/Consultant|THERA Technologies: Grant/Research Support|ViiV healthcare: Advisor/Consultant|ViiV healthcare: Grant/Research Support|ViiV healthcare: Honoraria **John R. Koethe, MD**, Gilead Sciences: Advisor/Consultant|Gilead Sciences: Grant/Research Support|Merck & Co.: Advisor/Consultant|Merck & Co.: Grant/Research Support **Jordan Lake, MD**, Merck: Advisor/Consultant|Theratechnologies: Advisor/Consultant **Mohammed Zogheib, PharmD, MPH**, Theratechnologies: Employee **Colleen McGary, PhD**, Theratechnologies: Employee **R Brandon Cash, PharmD**, Theratechnologies: Employee

